# Fusicoccin-A Targets Cancerous Inhibitor of Protein Phosphatase 2A by Stabilizing a C-Terminal Interaction with 14-3-3

**DOI:** 10.1021/acschembio.2c00299

**Published:** 2022-10-18

**Authors:** Hendrik J. Brink, Jeffrey R. van Senten, Ingrid J. De Vries-van Leeuwen, Daniel da Costa Pereira, Sander R. Piersma, Connie R. Jimenez, Federica Centorrino, Christian Ottmann, Marco Siderius, Martine J. Smit, Albertus H. de Boer

**Affiliations:** † Amsterdam Institute for Molecular and Life Sciences (AIMMS), Division of Medicinal Chemistry, Faculty of Sciences, Vrije Universiteit, De Boelelaan 1108, Amsterdam 1081 HZ, The Netherlands; ‡ OncoProteomics Laboratory, Department of Medical Oncology, Amsterdam University Medical Center (VUmc), 1081 HV Amsterdam, The Netherlands; § Laboratory of Chemical Biology, Department of Biomedical Engineering and Institute for Complex Molecular Systems (ICMS), Eindhoven University of Technology, 5600 MB Eindhoven, The Netherlands

## Abstract

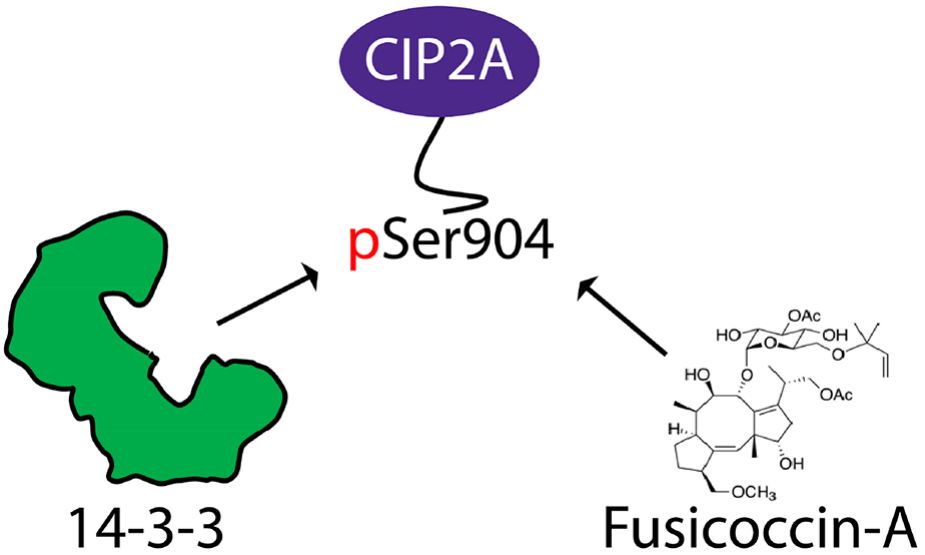

The cancerous inhibitor of protein phosphatase 2A (CIP2A) is an oncoprotein found overexpressed in many types of cancer. CIP2A has been shown to stabilize oncoproteins such as cMYC by shielding them from PP2A-mediated dephosphorylation. Here we report that the penultimate residue Ser904 in the C-terminus of CIP2A can be phosphorylated to create a binding site for the regulatory protein 14-3-3. We demonstrate that 14-3-3 is a new interaction partner of CIP2A. The 14-3-3/CIP2A C-terminal interaction complex can be targeted by the protein–protein interaction (PPI) stabilizer fusicoccin-A (FC-A), resulting in enhanced levels of phosphorylated Ser904. FC-A treatment of TNBC cells leads to the increased association of CIP2A with 14-3-3. We show that the composite interface between 14 and 3-3 and CIP2A’s C-terminus can be targeted by the PPI stabilizer FC-A, providing a new interface that could potentially be exploited to modulate CIP2A’s activity.

## Introduction

Breast cancer is the most frequently occurring cancer type in women worldwide.^1^ An oncoprotein found overexpressed in almost all cancer types, including breast cancer, is a cancerous inhibitor of protein phosphatase 2A (CIP2A).^2^ CIP2A overexpression has been correlated with a poor clinical outcome and a resistance to treatment.^3−7^ Specifically, in breast cancer, CIP2A overexpression was associated with tumor aggressiveness and the promotion of malignant growth.^8^ CIP2A has primarily been characterized in cancer cells as a direct inhibitor of protein phosphatase 2A (PP2A). In this role, CIP2A functions by binding to the B56 family of regulatory subunits from the heterotrimeric PP2A enzyme complex.^9^ As a result, CIP2A can activate oncogenic PP2A targets such as E2F1, AKT, and cMYC by protecting them from PP2A-mediated dephosphorylation.^10^ In a broader context, these findings support the notion that CIP2A is a clinically relevant oncoprotein in TNBC and thus an attractive therapeutic target.

It has been shown that polo-like kinase 1 (PLK1) can bind to the C-terminal tip of CIP2A and phosphorylate its penultimate residue Ser904.^11^ We hypothesized that phosphorylation of Ser904 could create a C-terminal binding site for the regulatory proteins 14-3-3, which act as molecular scaffolds in the cell through regulation of protein–protein interactions (PPIs) in key cellular processes. A unique feature of a subset of C-terminal 14-3-3 interactions is that they can be targeted by the diterpene glucoside Fusicoccin-A (FC-A) for PPI stabilization.^12,13^ In several studies, FC-A and its derivatives have been shown to have antiproliferative effects in cancer cells, including breast cancer.^14−16^

The development of small molecules that inhibit protein–protein interactions (PPIs) has proven to be a highly successful strategy in drug development.^17^ Modulating PPIs by so-called “molecular glues” that stabilize rather than inhibit PPIs is an emerging field in the drug discovery landscape.^18−20^ In contrast to PPI inhibition, PPI stabilizers such as FC-A target the composite interface between 14-3-3 and an interacting client protein, creating opportunities for enhanced specificity as the binding interface only exists within the context of the complex.

Here we report that phosphorylation of Ser904 creates a mode-III 14-3-3 binding site in the C-terminus of CIP2A. We show that the phosphorylated C-terminus of CIP2A can directly interact with 14-3-3. The composite interface between 14-3-3 and CIP2A can be targeted by the PPI stabilizer FC-A, which enhances phosphorylation levels of Ser904 and increases the association of 14-3-3 with CIP2A. With this study, we introduce a new player in the regulation of CIP2A mediated by C-terminal phosphorylation of residue Ser904, enabling binding of 14-3-3. Furthermore, we provide evidence for a small molecule PPI stabilizer targeting the composite interface between CIP2A and 14-3-3, potentially opening the door for novel ways to modulate CIP2A.

## Results & Discussion

### CIP2A’s C-Terminus Can Interact with 14-3-3η *In Vitro* and Is Sensitive to FC-A Stabilization

**Figure 1:**
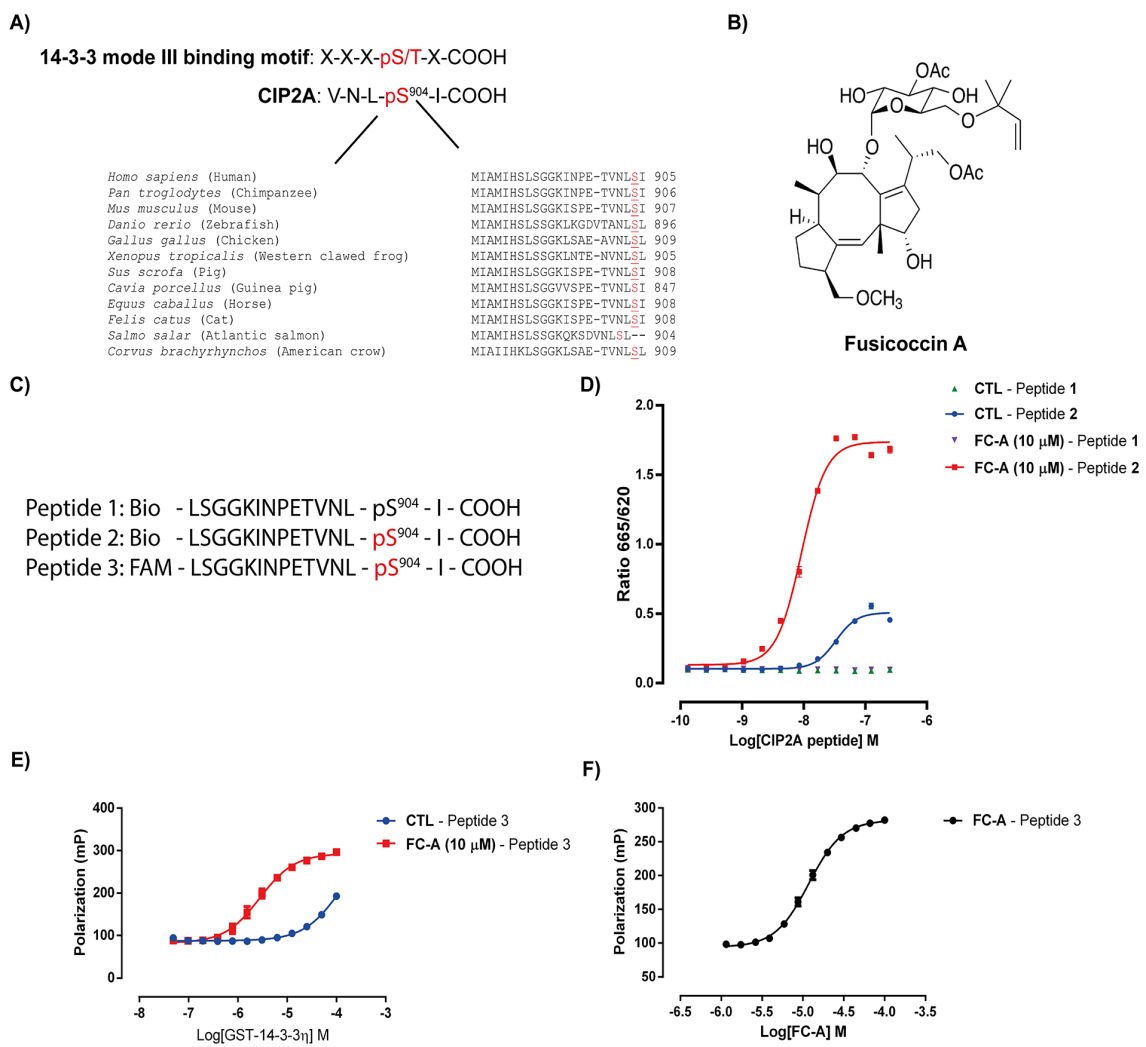
The C-terminus of CIP2A contains a mode-III 14-3-3 binding motif. (A) CIP2A’s C-terminus overlaid with 14-3-3 mode-III binding motif. The putative phosphosite is highlighted red with an alignment of the C-terminal amino acid sequence of CIP2A from different species shown below. (B) Chemical structure of Fusicoccin-A. (C) Overview of N-terminally biotinylated or fluorescein labeled CIP2A peptides either non- or phosphorylated on Ser904. (D) Homogeneous time-resolved FRET (HTRF) assay in which an N-terminally GST tagged 14-3-3η is targeted with an anti-GST europium (Eu) cryptate labeled monoclonal antibody and CIP2A peptides 1 and 2 used are N-terminally biotinylated and labeled with streptavidin conjugated to XL665. Concentration–response curves are shown for peptide 1 (Ser904) and peptide 2 (pSer904) in the presence or absence of a fixed 10 μM FC-A and 10 nM GST14-3-3η. Data is shown as mean ± SEM of three independent experiments. (E) Fluorescent polarization GST14-3-3η concentration–response curves generated in the presence of 10 μM FC-A or buffer using 100 nM of peptide 3. (F) Fluorescent polarization concentration–response curve of FC-A stabilizing the interaction between 1.5 μM GST14-3-3η and 100 nM peptide 3. Data is presented as the mean ± SEM of three independent experiments.

CIP2A was initially identified by mass spectrometry in an FC-A beads pulldown experiment using cell lysates from estrogen receptor alpha (ERα) positive MCF-7 breast cancer cells (see supplementary info Table 1). Studying the C-terminal amino acid sequence of CIP2A reveals that it contains a putative mode-III 14-3-3 interaction motif (x-x-x-[pS/pT]-X_1–2_-COOH), in which phosphorylation of Ser904 is a prerequisite for 14-3-3 binding. Sequence alignment of CIP2A showed that the extreme C-terminal tail is evolutionarily conserved among different species, suggesting a functional relevance (Figure 1A). Multiple phosphoproteomic studies have described CIP2A Ser904 as phosphorylated *in vitro*.^21,22^ Recently, Wang et al. showed that recombinant PLK1 could phosphorylate this site *in vitro*.^11^ Therefore, using (non)phosphorylated C-terminal CIP2A peptides, we investigated whether 14-3-3 could directly interact with CIP2A (Figure 1C). First, we developed a homogeneous time-resolved FRET (HTRF) assay for this putative interaction and its phosphorylation dependence (Figure 1D). The HTRF assay consisted of an N-terminally GST tagged 14-3-3η, which was targeted with an anti-GST europium (Eu) cryptate labeled monoclonal antibody and two (non)phosphorylated CIP2A peptides (peptide 1: Bio-LSSGGKINPETVNL-S^904^-I-COOH; peptide 2: Bio-LSSGGKINPETVNL-S^pS904^-I-COOH) that were N-terminally biotinylated and labeled with streptavidin conjugated to XL665. Europium is excited at a wavelength of 337 nm resulting in emission at a wavelength of 620 nm. If 14-3-3 interacts with the CIP2A peptide, resonance energy transfer can occur resulting in excitation and emission of XL665 at a wavelength of 665 nm. Concentration–response curves generated with peptide 2 revealed that the C-terminal tip of CIP2A can directly interact with 14-3-3 with an EC_50_ value of 47 nM and that this interaction is sensitive to FC-A-mediated (Figure 1B) stabilization resulting in an EC_50_ shift to 9.33 nM (Figure 1D). Additionally, concentration–response curves generated with peptide 1 confirm that the interaction is phosphorylation-dependent, as the nonphosphorylated peptide does not interact with 14-3-3 (Figure 1D).

Next, we used an orthogonal fluorescent polarization (FP) assay to confirm the results obtained with our HTRF assay. First, concentration–response curves were made using peptide 3, which was N-terminally labeled with fluorescein and in which Ser904 is phosphorylated by titrating to N-terminally GST-tagged 14-3-3η (GST-14-3-3η) at a fixed concentration of FC-A (10 μM) or a buffer control. The results demonstrate that GST-14-3-3η can bind to the FAM-pCIP2A peptide. However, the affinity of the complex is low, with an EC_50_ value of 118 μM (Figure 1E). Targeting the 14-3-3η/pCIP2A complex with the PPI stabilizer FC-A greatly enhanced the affinity of the interaction, resulting in an EC_50_ value shift to 2.76 μM (Figure 1F). Next, FC-A concentration–response curves were generated showing enhanced binding of 14-3-3η to pCIP2A (EC_50_ value of 11.8 μM), further supporting the idea that the 14-3-3η/pCIP2A complex can be targeted by FC-A (Figure 1F). Thus, *in vitro* 14-3-3 can bind directly to the C-terminal tail of CIP2A in a phosphorylation-dependent manner, and FC-A stabilizes this interaction.

### Cocrystallization of CIP2A’s Phosphorylated C-Terminus with 14-3-3/FC-A

**Figure 2:**
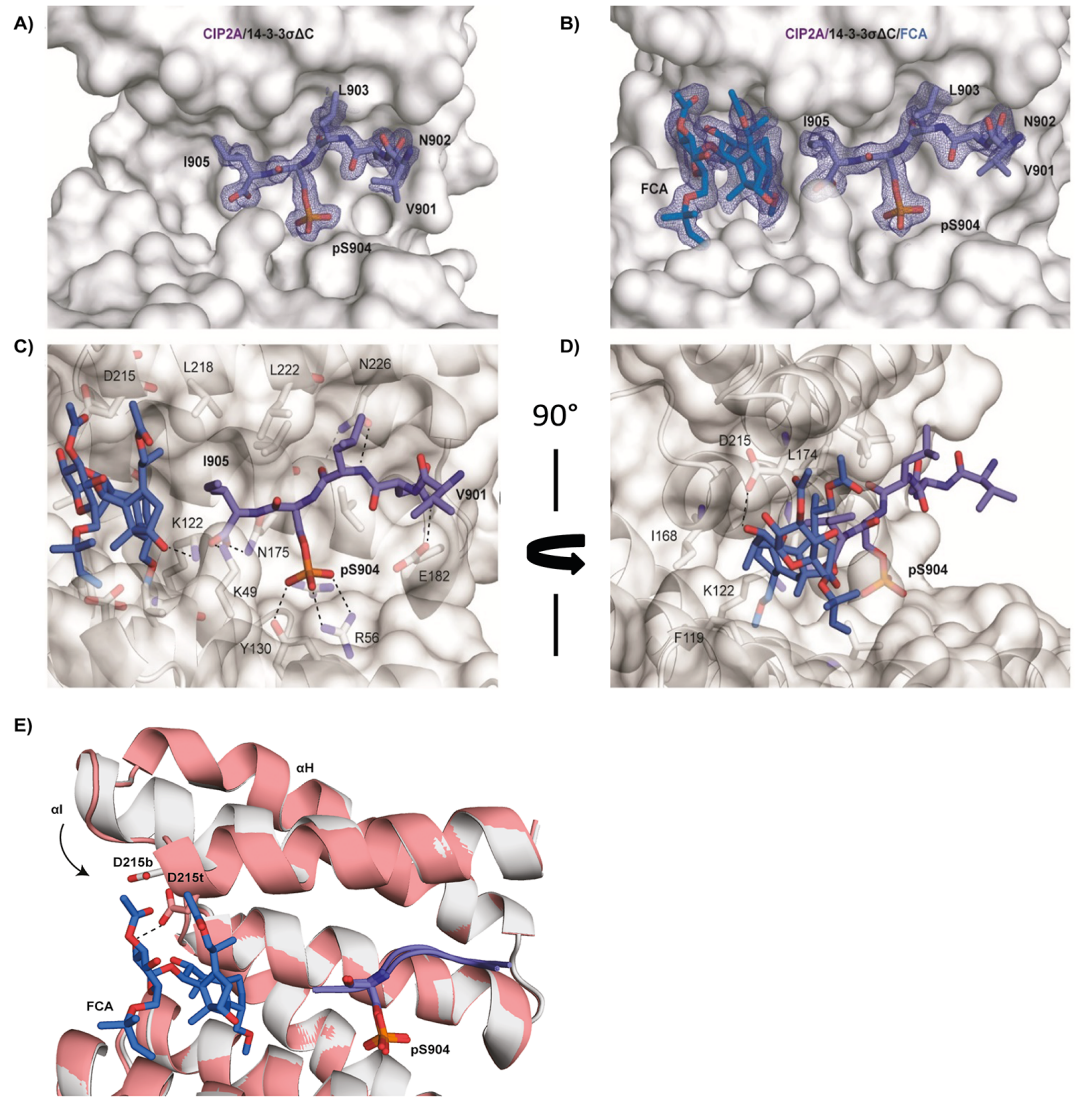
Crystal structure of CIP2A/14-3-3σΔC and CIP2A/14-3-3σΔC/FC-A. (A) Crystal structure (resolution 1.8 Å) of the CIP2A C-terminus peptide (sequence: ^895^KINPETVNL{pS}I^905^) in complex with 14-3-3σΔC protein. (B) Crystal structure (resolution 2.0 Å) of the protein/peptide/FC-A complex. 14-3-3σΔC is displayed as white surface or cartoon. The CIP2A peptide and FC-A are depicted with purple or blue sticks, respectively. The 2F_0_-F_C_ density map for CIP2A peptide and FC-A is contoured at 1σ. (C,D) Front and side view of the ternary complex. Polar contacts are depicted as black dotted lines. (E) Alignment of the high-resolution crystal structures of the binary complex 14-3-3σΔC/CIP2A (PDB ID: 7BM9) and ternary complex 14-3-3σΔC/CIP2A/FCA (PDB ID: 7BMC). The structure of 14-3-3 is depicted as a gray cartoon in the binary complex and a pink cartoon in the ternary.

For structural analysis of the interaction, a CIP2A peptide representing the C-terminal amino acid sequence was cocrystallized with 14-3-3σΔC. The space group was determined to be C2221 with one 14-3-3 monomer in the asymmetric unit. The 2F_0_-F_C_ density map allowed us to build five amino acids of the peptide sequence (Figure 2A). Analysis of the crystal structure reveals a typical mode-III binding motif. The binding of the peptide is primarily mediated by the polar contacts between Lys49, Arg56, Arg129, and Tyr130, consistent with most 14-3-3 binding partners. Additional polar contacts are mediated by Asn175, Glu182, and Asn226 (Figure 2C). At the C-terminus, the side chain of Ile905 is oriented toward the hydrophobic region of the amphipathic groove, which is known to accommodate FC-A. To explain the stabilization effect displayed on the interaction by FC-A, the compound was soaked in the crystals of the binary complex. Additional electron density could be observed that allowed us to clearly build the FC-A structure in the complex (Figure 2B). The FC-A molecule is deeply buried in the hydrophobic groove where amino acids Val46, Phe119, Ile168, Leu218, Ile219, and Leu222 interact with the diterpene moiety of the scaffold. Polar contacts are established between FC-A and Lys122 and Asp215. The most prominent interaction between FC-A and CIP2A peptide is represented by the hydrophobic contact of the C-terminal Ile905 of the peptide, which is directly aligned with the FC-A molecule (Figure 2C,D). In Figure 2E, an alignment of the crystal structures of the binary complex 14-3-3σΔC/CIP2A and ternary complex 14-3-3σΔC/CIP2A/FCA is shown. From the crystallographic alignment, it is possible to observe the conformational change induced to helix αI in the ternary complex and driven by the polar contact between D215 and FC-A. D215 is highlighted with gray sticks in the binary complex (D215b) and pink sticks in the ternary complex (D215t) (Figure 2E).

### CIP2A Binds to 14-3-3 in a Phosphorylation-Dependent Manner

**Figure 3:**
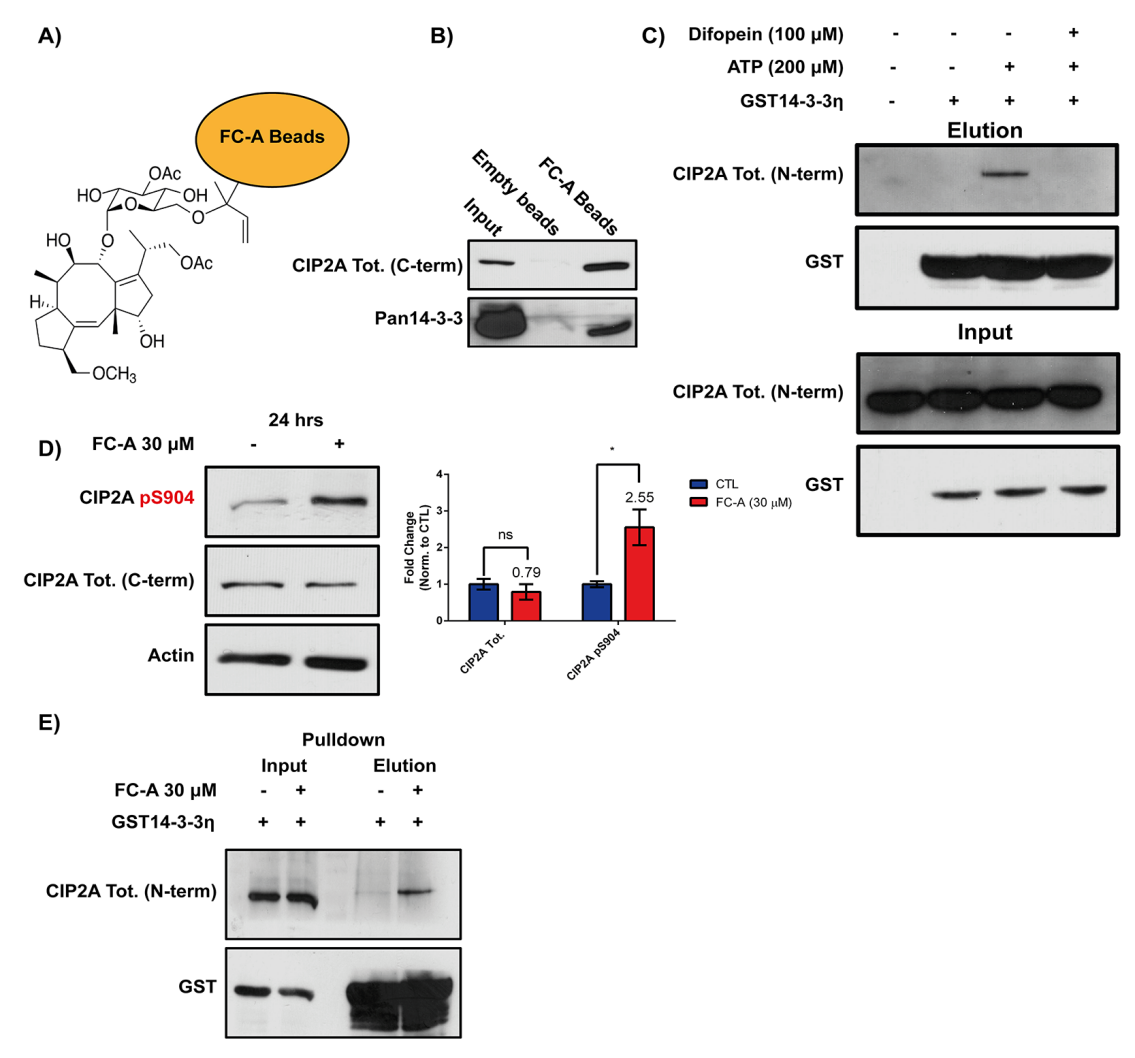
Full-length CIP2A can interact with 14-3-3 *in vitro*. (A) Cartoon showing FC-A coupled to magnetic hydrazide beads. (B) Western blot showing the results of an FC-beads pulldown experiment in MDA-MB-468 cell lysates. (C) Western blot of the GST14-3-3η pulldown of CIP2A in MDA-MB-468 cell lysates. The top panel shows pulldown elution samples, and the bottom panel shows input samples of each pulldown. (D) Western blot of MDA-MB-468 cell lysates after treatment of cells with 30 μM FC-A or a vehicle control for 24 h before harvest. Quantification was performed after normalization to an actin loading control. Data are presented as mean ± SEM and analyzed with an unpaired *t* test corrected for multiple comparisons with the Holm-Sidak method *P = 0.036. Membrane was probed with our custom CIP2A pS904, C-terminal CIP2A, and actin antibodies. (E) Western blot of GST14-3-3η pulldown of CIP2A in MDA-MB-468 cell lysates from cells either treated with 30 μM FC-A or a vehicle control for 24 h before harvest. Representative Western blots are shown of three (B–D) or two (E) independent experiments.

Cocrystallization of the pCIP2A peptide with 14-3-3/FC-A revealed that CIP2A could interact directly with 14-3-3 in a mode-III manner. Consequently, we wanted to interrogate this interaction with full-length proteins further. CIP2A was initially found in an FC-A beads pulldown in MCF-7 cells. However, in these cells, CIP2A is under transcriptional control of ERα, a known 14-3-3/FC-A target.^14,23^ Therefore, we first decided to test whether the 14-3-3/FC-A/CIP2A complex can be identified in TNBC cells lacking ERα. FC-A was coupled to magnetic hydrazide beads and used for affinity pulldown experiments using the TNBC cell line MDA-MB-468 (Figure 3A). Western blots of the pulldown on cell lysates revealed that CIP2A was present in a complex with 14-3-3/FC-A, while empty hydrazide beads lacking FC-A were unable to pulldown CIP2A or 14-3-3 (Figure 3B). GST-14-3-3η pulldown experiments confirmed the results obtained with FC-A beads, as CIP2A could be found associated with 14-3-3 (Figure 3C). The interaction between CIP2A and 14-3-3 appeared to be phosphorylation-dependent, as more CIP2A was identified in pulldown samples that had been preincubated with ATP at 37 °C (Figure 3C). No CIP2A was identified in the sample lacking GST-14-3-3η. In addition, blocking 14-3-3’s amphipathic binding groove with the nonphosphorylated competing peptide difopein, which binds 14-3-3 with a high affinity, yielded similar results. Taken together, these findings show that CIP2A is present in a complex with 14-3-3. Furthermore, the association of CIP2A with 14-3-3 appears to be phosphorylation-dependent.

### FC-A Enhances CIP2A Ser904 Phosphorylation in Cells

Stimulation of phosphorylation by incubating the cell lysates with ATP at 37 °C was required to pulldown CIP2A, indicating phosphorylation of CIP2A is required for complex formation with 14-3-3. Therefore, we decided to study the effect of 14-3-3 binding on the phosphorylation status of CIP2A’s C-terminus. A phosphorylation-specific antibody against CIP2A Ser904 was generated using a phosphorylated C-terminal peptide (LSGGKINPETVNL-pS^904^-I-COOH), and specificity was verified using dot blots, dephosphorylation of the CIP2A endogenous protein and comparing detection of exogenously expressed CIP2A WT or a C-terminal S904A mutant (Figure S1A–H). Treatment of MDA-MB-468 cells with FC-A for 24 h did not affect total CIP2A, but Ser904 phosphorylation levels were enhanced (Figure 3D). Similarly, treatment of MDA-MB-468 cells with cycloheximide and FC-A showed no changes in total CIP2A over a 24 h period (Figure S1I,J). These findings indicate that CIP2A total protein levels are not affected by FC-A in the time frame that we investigated changes in Ser904 phosphorylation levels. Subsequently, GST-14-3-3η pulldown experiments on FC-A treated cells revealed that more CIP2A could be detected in these samples compared to control cell lysates (Figure 3E). These results demonstrate that FC-A treatment of cells enhances CIP2A Ser904 phosphorylation levels, resulting in more CIP2A found in complex with 14-3-3. Likewise, De Vries-van Leeuwen et al. showed that treating MCF-7 cells with FC-A is necessary to detect C-terminal phosphorylation of the ERα.^14^ In the case of the ERα, however, the treatment of cells with the proteasome inhibitor MG132 is an additional requirement for phosphorylation of the C-terminal tip, indicating a possible role for 14-3-3 in ERα degradation. In contrast, increased phosphorylation of Ser904 did not affect total CIP2A levels in the first 24 h after FC-A treatment indicating that Ser904 phosphorylation is not directly linked to CIP2A turnover. The ability of FC-A to increase CIP2A’s pSer904 levels may be the result of an increased affinity between CIP2A and 14-3-3, resulting in shielding of the C-terminal tip of CIP2A from phosphatases by the 14-3-3/FC-A complex. A similar observation was made for the H^+^-ATPase in plants, in which 14-3-3/FC-A shields the penultimate C-terminal phosphorylation site from phosphatases.^24^ Taken together, we show that CIP2A can be targeted with a small molecule at the protein level, modulating its C-terminal phosphorylation status and regulating the interaction with 14-3-3.

### FC-A Targets the Composite Interface between CIP2A and 14-3-3

CIP2A is an attractive therapeutic target considering its role in directly binding and inhibiting PP2A while stabilizing its oncogenic partners such as cMYC, E2F1, AKT, and β-catenin.^10,25^ Nonetheless, only a limited number of small molecules are known that modulate CIP2A activity. Here we identify a small molecule that targets the composite interface between CIP2A and 14-3-3 in a noncovalent manner. We show that FC-A-mediated 14-3-3 binding enhances Ser904 phosphorylation. As such, our work potentially opens up new avenues for targeting CIP2A in cancer. Approaches can be taken to identify small molecules that target the druggable interface between CIP2A and 14-3-3, which can be aided by the crystal structures provided in this study. While further studies are required to understand how 14-3-3 modulates CIP2A’s activity, it can be envisaged that using a molecular “glue” such as FC-A to stick 14-3-3 to the C-terminus of CIP2A could be a novel strategy to modulate CIP2A’s interactome. In this setting, 14-3-3 could potentially block CIP2A’s interactions with oncoproteins such as cMYC, similar to what we have previously shown for the ERα.^21^

In this context, there have recently been several promising advances in identifying and developing PPI stabilizers that specifically target a particular 14-3-3 interaction, such as that of 14-3-3 and ERα. Sijbesma et al. recently developed a site-directed fragment-based screening platform using a fragment tethering approach to identify novel orthosteric stabilizers targeting the 14-3-3/ERα interaction interface.^26^ Moreover, work from the same group illustrated that the semisynthetic FC derivative DP-005 could target the interaction between 14-3-3/p65 and was 10-fold more active in stabilizing this particular interaction than any other 14-3-3 PPI tested in their study.^27^ These findings illustrate that small modifications of PPI stabilizers can have large impacts on their specificity, which could allow for the development of potent and selective PPI stabilizers targeting 14-3-3/CIP2A in cancer.

## Supporting Information Available

Proteins identified in FC-Beads pulldown by mass spectrometry (Table S1), XRD data collection and refinement statistics (Table S2), custom CIP2A pSer904 antibody validation data, cyclohexmide treatment data (Figure S1), experimental methods (PDF)
