# Supplementary material for: Fusicoccin-A Targets Cancerous Inhibitor of Protein Phosphatase 2A by Stabilizing a C-Terminal Interaction with 14-3-3
Source: ACS Chem Biol. 2022 Oct 18;17(11):2972–8. doi: 10.1021/acschembio.2c00299 (PMC9679992; doi:10.1021/acschembio.2c00299)
Supplement: Supplementary file 1 — cb2c00299_si_001.pdf [file cb2c00299_si_001.pdf]

Supporting information

Fusicoccin-A targets cancerous inhibitor of protein phosphatase 2A by stabilizing a C-terminal interaction with 14-3-3

Hendrik J. Brink[1], Jeffrey R. van Senten[1], Ingrid J. De Vries-van Leeuwen[1], Daniel da Costa Pereira[1], Sander R. Piersma[2], Connie R. Jimenez[2], Federica Centorrino[3], Christian Ottmann[3], Marco Siderius[1], Martine J. Smit[1], *Albertus H. de Boer[1]

1. Amsterdam Institute for Molecular and Life Sciences (AIMMS), Division of Medicinal Chemistry, Faculty of Sciences, Vrije Universiteit, De Boelelaan 1108, Amsterdam 1081 HZ, The Netherlands
2. OncoProteomics Laboratory, Department of Medical Oncology, Amsterdam University Medical Center (VUmc), 1081 HV Amsterdam, The Netherlands
3. Laboratory of Chemical Biology, Department of Biomedical Engineering and Institute for Complex Molecular Systems (ICMS), Eindhoven University of Technology, 5600 MB Eindhoven, The Netherlands.

Correspondence to: ***Albertus H. de Boer, **email: a.h.de.boer@vu.nl

Index

Table S1 Proteins identified in FC-Beads pulldown by mass spectrometry. 3

Table S2. Data collection and refinement statistics for X-ray crystallization. 4

Supplemental info figure 1 Custom CIP2A pS904 antibody specifically recognizes the pCIP2A peptide. 6

Material and Methods 7

Protein expression and purification for X-ray Crystallography 7

Protein crystallization and structure elucidation 7

FC-A treatment of cells and cell lysate preparation 7

Fluorescent Polarization 8

FC-A Beads production 8

FC-Beads pulldown 8

Mass spectrometry analysis (nano-LC/MS-MS). 8

Western blot 9

GST14-3-3η protein production and purification 10

GST-14-3-3η pulldowns 10

Quantification and statistical analysis 10

Data and code availability 11

Table S1 Proteins identified in FC-Beads pulldown by mass spectrometry.

Selected proteins identified in a FC-Beads pulldown of MCF-7 cell lysates that contain a putative mode-III 14-3-3 binding site on their C-terminal tip, the last five amino acids of each protein are shown. Pulldowns were eluted with a non-interacting peptide (NIP-2) or the R18 peptide that specifically binds 14-3-3. The number of assigned spectra are shown and proteins are sorted based on their ratio of assigned spectra (NIP-2/FC-Bead spectra divided over the R18/FC-Bead spectra).

No.	C-term tip	Identified proteins (20)	Uniprot Accession	NIP-2 + FC-beads	R18 + FC-Beads	Ratios
1	EGTSV	Isoform 3 of Ribosome-binding protein 1	Q9P2E9	0	4	0.0
2	**VNLSI**	**Isoform 1 of Protein CIP2A**	**Q8TCG1**	**0**	**2**	**0.0**
3	SKKTV	Dolichyl-diphosphooligosaccharide--protein glycosyltransferase subunit STT3B	Q8TCJ2	0	2	0.0
4	KEPSL	Tumor-associated calcium signal transducer 2	P09758	4	1	0.3
5	KFYSL	Pre-mRNA-processing factor 19	Q9UMS4	30	17	0.6
6	MSHSL	Mitochondrial 28S ribosomal protein S2	Q9Y399	3	2	0.7
7	VGTSV	Lysyl-tRNA synthetase	Q15046	46	42	0.9
8	VQESV	40S ribosomal protein S3a	P61247	13	12	0.9
9	SKSTI	Isoform 1 of Polypyrimidine tract-binding protein 1	P26599	7	7	1.0
10	LQQSL	Mitochondrial import inner membrane translocase subunit Tim23	O14925	3	3	1.0
11	PVFTL	Cytochrome c oxidase subunit 2	P00403	6	7	1.2
12	GVPTV	Isoform 1 of Polyadenylate-binding protein 1	P11940	41	51	1.2
13	MMNTV	Isoform Alpha of Signal transducer and activator of transcription 1-alpha/beta	P42224	12	16	1.3
14	SPPTV	Puromycin-sensitive aminopeptidase	P55786	15	21	1.4
15	QPWSV	6-phosphofructokinase type C	Q01813	1	2	2.0
16	LETSL	Isoform D of Plasma membrane calcium-transporting ATPase 1	P20020	1	2	2.0
17	MGTSL	cDNA FLJ56403, highly similar to CDK5 regulatory subunit-associated protein 3	Q96JB5	3	7	2.3
18	KKTSI	Coatomer subunit beta	P53618	1	4	4.0
19	SLPSL	Isoform 1 of Rab3 GTPase-activating protein non-catalytic subunit	Q9H2M9	1	6	6.0
20	QETSL	Sodium-dependent multivitamin transporter	Q9Y289	1	7	7.0

Table S2 Data collection and refinement statistics for X-ray crystallization.
Data collection statistics were calculated with Aimless and refinement statistics were extracted using the "table 1" tool of Phenix. Values in parenthesis correlate to high resolution shell.

	14-3-3σΔC /CIP2A	14-3-3σΔC /CIP2A/FC-A
**PDB ID**	7BM9	7BMC
**Data collection**		
Wavelength (Å)	1.5419	1.5419
Resolution (Å)	41.86 - 1.80 (1.83 - 1.80)	31.30 - 2.00 (2.04 - 2.00)
Space group	C2221	C2221
Unit cell (Å)	82.55 112.32 62.81	83.72 111.43 62.61
Total reflections	169094 (6511)	62670 (2971)
Unique reflections	26877 (1251)	19805 (927)
Multiplicity	6.3 (5.2)	3.2 (3.2)
Completeness (%)	98.2 (92.7)	98.6 (92.2)
Average I/σ	15.5 (4.8)	6.5 (3.3)
Wilson B-factor	8.45	10.58
R-merge	0.092 (0.305)	0.098 (0.267)
R-meas	0.100 (0.336)	0.119 (0.321)
$CC_{1/2}$	0.997 (0.929)	0.955 (0.903)
**Refinement**		
Number of protein/ solvent/ligand atoms	1929/510/3	1884/348/54
R_{work}/R_{free} (%)	13.92/18.65	18.32/22.02
RMSD Bond lengths (Å)	0.009	0.004
RMSD Bond angles (°)	1.13	0.87
Ramachandran favored (%)	98.28	98.67
Ramachandran allowed (%)	1.72	1.33
Ramachandran outliers (%)	0.00	0.00
Rotamer outliers (%)	0.49	0.00
Clashscore	4.18	1.85
Average B-factor	12.95	15.97

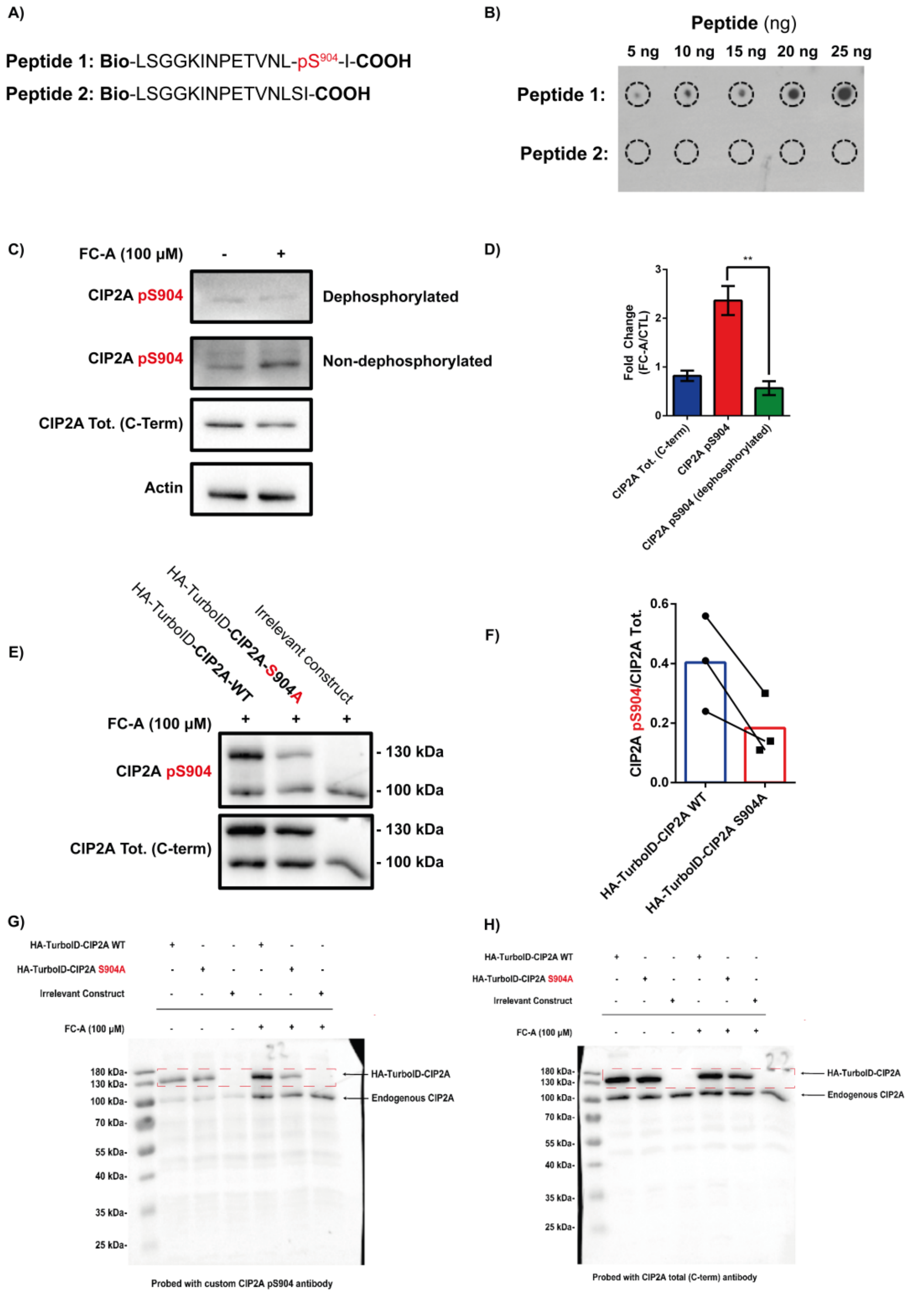

A)
Peptide 1: Bio-LSGGKINPETVNL-pS904-I-COOH
Peptide 2: Bio-LSGGKINPETVNLSI-COOH
B)
Peptide (ng)
5 ng
10 ng
15 ng
20 ng
25 ng
Peptide 1:
Peptide 2:
C)
FC-A (100 μM)
CIP2A pS904
Dephosphorylated
CIP2A pS904
Non-dephosphorylated
CIP2A Tot. (C-Term)
Actin
D)
Fold Change (FC-A/CTL)
CIP2A Tot. (C-term)
CIP2A pS904
CIP2A pS904 (dephosphorylated)
E)
HA-TurboID-CIP2A-WT
HA-TurboID-CIP2A-S904A
Irrelevant construct
FC-A (100 μM)
CIP2A pS904
CIP2A Tot. (C-term)
130 kDa
100 kDa
F)
CIP2A pS904/CIP2A Tot.
HA-TurboID-CIP2A WT
HA-TurboID-CIP2A S904A
G)
HA-TurboID-CIP2A WT
HA-TurboID-CIP2A S904A
Irrelevant Construct
FC-A (100 μM)
HA-TurboID-CIP2A
Endogenous CIP2A
Probed with custom CIP2A pS904 antibody
H)
HA-TurboID-CIP2A WT
HA-TurboID-CIP2A S904A
Irrelevant Construct
FC-A (100 μM)
HA-TurboID-CIP2A
Endogenous CIP2A
Probed with CIP2A total (C-term) antibody

H)

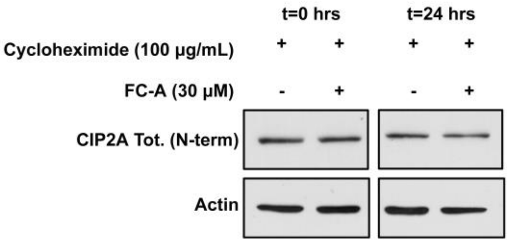

I)

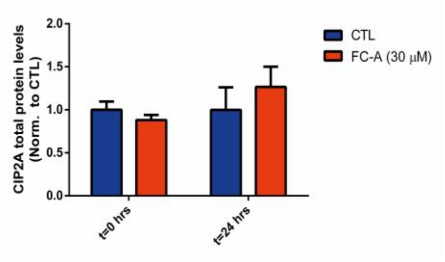

Figure S1 Custom CIP2A pS904 antibody specifically recognizes the pCIP2A peptide. A) Overview of peptides used in this study to generate the custom phosphorylation specific CIP2A antibody targeting Ser904. **B)** Dot blot of membrane on which peptides 1 and 2 had been spotted in increasing amounts that were either unphosphorylated or phosphorylated on Ser904. **C)** Western blot of HEK293T cell lysates derived from cells treated with 100 µM FC-A or a vehicle control for 24 hours. CIP2A was dephosphorylated on the PVDF membrane with FastAP after transfer and subsequently visualized with CIP2A total (C-term; sc-80659) and custom CIP2A pS904 antibodies. Actin is shown as a loading control. A representative western blot is shown of three independent experiments. **D)** Quantification of western blot shown in C) data is presented as mean ± SEM and analyzed with an unpaired t-test corrected for multiple comparisons with the Holm-Sidak method **P=0.005. **E)** Western blot of HEK293T cells transiently transfected with either HA-TurboID-CIP2A WT or S904A mutant or an irrelevant construct. HEK293T cells had been incubated with 100 µM FC-A for 24 hours post transfection. Membrane was analysed with CIP2A total (C-term; sc-80659) and custom CIP2A pS904 antibody. A representative western blot is shown of three independent experiments. **F)** Quantification of western blot data shown in E. Total membranes of data shown in E are displayed in **G)** & **H)**. Data is presented as mean ± SEM and analyzed with an unpaired t-test corrected for multiple comparisons with the Holm-Sidak method P=0.11. **I)** Western blot of MDA-MB-468 cells that had been treated with 100 µg/mL cycloheximide for 24 hrs in the absence or presence of 30 µM FC-A. Membrane analysed with CIP2A total (N-term; Cell Signalling, #14805) and beta actin (Sigma, Cat#A5316) antibodies. **J)** Quantification of data shown in I from three independent experiments.

Material and Methods

Protein expression and purification for X-ray Crystallography

14-3-3σΔC (C-terminally truncated after T231) was expressed in BL21 (DE3) competent cells via a pProEX HTb plasmid. Expression was induced with 0.4 mM Isopropyl β-D-1-thiogalactopyranoside (IPTG) overnight at 18°C. After spinning down and lysis of the expression culture, the protein was purified on a Ni2+ -NTA columns. The His6-tag was cleaved with Tobacco Etch Virus (TEV) protease in 1:0.05 mg ratio and a second nickel-affinity chromatography was performed followed by size exclusion chromatography (Superdex75) in 25 mM Hepes pH 7.5, 100 mM NaCl, 10 mM MgCl2, 2 mM β-mercaptoethanol.

Protein crystallization and structure elucidation

Crystals of the 14-3-3σΔC /CIP2ApS904 complex were grown by mixing 10 mg·mL^{-1} 14-3-3σΔC in a molar ratio of 1:2 with the peptide, in 20 mM Hepes pH 7.5, 2 mM $MgCl_2$, and 2 mM β-mercaptoethanol, and incubated overnight at 4°C. The complex was then set up for crystallization using the sitting drop method by mixing 1:1 with the precipitation buffer 0.095 M Hepes pH 7.1, 25% PEG 400, 0.19 M $CaCl_2$ and 5% Glycerol. Crystals were grown within 7 days and could be directly flash-cooled in liquid nitrogen. For the soaking, a 10 mM stock solution in DMSO of FC-A was added to the crystals to a final concentration of 1mM. Crystals were harvested after 5 days incubation and directly flash-cooled in liquid nitrogen. Data collection was performed in house on a Rigaku MicroMax-003 (Rigaku Europe, Kemsing Sevenoaks, UK) sealed tube X-ray source and a Rigaku Dectris PILATUS 200K detector (DECTRIS Ltd., Baden-Daettwil, Switzerland). Data were processed using DIALS (Winter *et al.*, 2018). Molecular replacement was carried out using Phaser (McCoy *et al.*, 2007) and PDB ID: 4JC3 as template. The obtained model was subjected to reiterative rounds of model building and refinement using Coot (Emsley and Cowtan, 2004) and PHENIX (Adams *et al.*, 2010). Figures were prepared using Pymol software (DeLano, 2002).

FC-A treatment of cells and cell lysate preparation

MDA-MB-468 (ATCC, Cat#HTB-132) cells were grown in DMEM (Dulbecco), supplemented with 10% fetal bovine serum (FBS, Gibco) and 1% (50 IU/mL) penicillin and streptomycin at 37°C with 5% CO_2 in a humidified atmosphere. For FC-A treatments cells were seeded and allowed to attach and grow for 24 hours before medium was refreshed containing FC-A or DMSO. Cells were grown in the presence of FC-A for 24 hours before harvest. Cells were harvested by aspirating medium followed by washing two times with chilled PBS (4°C). Subsequently, a modified NP-40 cell lysis buffer (Tris-HCl 50 mM, Glycerol 10%, NaCl 100 mM, EDTA 5 mM, $MgCl_2$ 2 mM, 1% NP-40, 0.1% SDS, 50 mM NaF, Cantharidin 10 µM, $NaVO_4$, pH=8.0 supplemented with PhosSTOP and protease inhibitor cocktail EDTA free from Roche) was added and incubated with cells for 5 minutes before cells were collected by scrapping and incubated on a rotor at 4°C for 10 minutes. In the case of FC-beads or GST-14-3-3η pulldowns SDS was omitted from the lysis buffer. Lysates were subsequently clarified by centrifuging for 10 minutes at 10 000 RCF at 4°C. Supernatants were collected and lysate concentrations were determined by BCA assay (Pierce, Cat#23225) per the manufacturer's protocol.

Fluorescent Polarization

N-terminal fluorescein (FAM) labelled phosphorylated peptides were purchased from GL Biochem. The C-terminal CIP2A peptide had the sequence FAM – I-N-P-E-T-V-N-L-(pS^{904})-I – COOH. The FAM - CIP2A peptide was dissolved in phosphate saline buffer (PBS) with a pH of 7.5. Concentration curves of recombinant GST-14-3-3η were produced by mixing various concentrations of the protein diluted in assay buffer (PBS, pH=7.5) with a fixed 100 nM of the FAM labelled peptide and either a DMSO vehicle control or a fixed 10 μM Fusicoccin – A (FC-A). FC-A concentration curves were generated by mixing various concentrations of FC-A diluted in PBS with a fixed 100 nM of FAM - CIP2A and a fixed 4.7 μM of GST-14-3-3η. Samples were added to sterile black 384 wells plate and incubated at room temperature (RT) for 30 minutes before polarization measurements were performed on the PHERAstar FS (BMG labtech GmbH, Ortenberg, Germany) using the FP 480 – 520 nm fixed polarization filter set.

FC-A Beads production

FC-A beads were produced in a two-step reaction as previously described by Feyerabend and Weiler, 1987 by first generating FC-aldehyde (FC-Ald) which, was subsequently coupled to magnetic hydrazide beads (Chemicell, Cat#1404). FC-A was oxidized by first dissolving it in tetrahydrofuran (THF) and subsequently incubated in the dark overnight (O/N) with OsO_4 and $NaIO_4$. The THF was evaporated using N_2 gas. The reaction product was purified using a Sep-Pak C18 (Millipore Waters) column, product was eluted with methanol (MeOH). Before coupling to magnetic hydrazide beads reaction efficiency was determined by HPLC analysis. Magnetic hydrazide beads were washed three times in PBS before incubating for 1 hour in the dark at RT with FC-Ald in coupling buffer consisting of 100 mM NaAc, 0.02% NaN_3 (pH 5.0, set with acetic acid). Subsequently, $NaCNBH_3$ was added to the coupling buffer and incubated stirring O/N at 4°C in the dark. Next, the FC-A beads were washed three times with PBS and empty sites were blocked with D-glyceraldehyde in PBS for 1 hour at RT whilst stirring. FC-A beads were finally washed ten times with PBS before storage in MeOH at a concentration of 50 mg/mL.

FC-Beads pulldown

FC-A beads pulldowns were performed using cell lysates from MDA-MB-468 (ATCC, Cat#HTB-132) cells prepared as described above. Empty control beads or FC-A beads were washed three times in 750 µL of tris buffered saline (TBS) pH=7.5 before being reconstituted in 50 µL of TBS and added to 500 ug of cell lysate and incubated on a rotor for 1 hour at RT. Next, the supernatant was removed and beads were washed three times with 1 mL of wash buffer (TBS-T) by gently inverting. Samples were subsequently eluted with 40 µL of 0.2% SDS followed by boiling in 50 µL of Laemmli's sample buffer at 96°C for 10 minutes for western blot analysis.

Mass spectrometry analysis (nano-LC/MS-MS).

Proteins in the FC-beads eluate were separated on an SDS-PAGE gel and visualized with coomassie. The SDS-PAGE gel was subdivided into small cubes and washed with 50 mM ammonium bicarbonate (50-ABC) and then with 50-ABC in 50% acetonitrile (50-ABC/ACN). Wash cycle was repeated twice. Proteins were subsequently reduced with 10 mM dithiothreitol (DTT) in 50-ABC for 1 hour at 37°C. After which, samples

were submitted to an alkylation with 55 mM iodoacetamide in 50-ABC for 45 min at RT in the dark. Samples were then washed two times with 50 mM ammonium bicarbonate (50-ABC) and followed by 50-ABC in 50% acetonitrile (50-ABC/ACN). After washing samples were dried in a speedvac. Gel cubes were then rehydrated in 50-ABC containing 6.25 ng/µl trypsin (Sequence grade modified trypsin; Promega) for 10 min. Digestion of samples took place O/N at 25°C. Peptides were subsequently extracted with 1% formic acid (FA) and then followed two extractions with 5% FA in 50% ACN. Extractions were subsequently pooled and stored at -20°C. Sample volumes were then reduced to 50 µl in a speedvac before injecting 10 µl into anUltimate 3000 nanoLC system (Dionex LC-Packings, Amsterdam, The Netherlands) equipped with a 20 cm x 75 µm ID fused silica column custom packed with 3 µm 120 Å ReproSil Pur C18 aqua (Dr Maisch GMBH, Ammerbuch-Entringen, Germany). Peptides were separated at 300 nl/min in a 10-40% buffer B gradient in 60 min. Peptide mass spectra were acquired on a LTQ-FT hybrid mass spectrometer (Thermo Fisher, Bremen, Germany). Intact masses were measured at resolution 50,000 in the ICR cell using a target value of 1 x 10^6 charges. In parallel, following an FT pre-scan, the top 5 peptide signals (charge-states 2+ and higher) were submitted to MS/MS in the linear ion trap (3 amu isolation width, 30 ms activation, 35% normalized activation energy, Q value of 0.25 and a threshold of 5,000 counts). Dynamic exclusion was applied with a repeat count of 1 and an exclusion time of 30 sec. MS/MS spectra were searched against the human IPI database 3.31 (67,511 entries) using Sequest (version 27, rev 12), which is part of the BioWorks 3.3 data analysis package (Thermo Fisher, San Jose, CA). MS/MS spectra were searched with a maximum allowed deviation of 10 ppm for the precursor mass and 1 amu for fragment masses. Methionine oxidation and cysteine carboxamidomethylation were allowed as variable modifications, two missed cleavages were allowed and the minimum number of tryptic termini was 1. After database searching the DTA and OUT files were imported into Scaffold 3 (Proteomesoftware, Portland, OR) to validate peptide identifications using the PeptideProphet algorithm, only identifications with a probability >95% were retained. Subsequently, the ProteinProphet algorithm was applied and protein identifications with a probability of >99% with 2 peptides or more in at least one of the samples were retained.

Western blot

Cell lysates were prepared as described above. Total protein levels were determined by BCA assay (Pierce, Cat#23225) and for western blots analysing protein changes after FC-A treatment 20 µg of total cell lysate was used. For GST-14-3-3η and FC-beads pulldown 20 µL of elution samples were used. Cell lysates were incubated in Laemmli's sample buffer at 96°C for 10 minutes before running on a 10% SDS-PAGE gel. Samples were transferred to a PVDF membrane at 4°C which, was subsequently blocked with either 5% skimmed milk powder (Sigma,Cat#70166) or 5% bovine serum albumin (BSA) (Melford, Cat#A30075) depending on the primary antibody used for 30 minutes on a shaker. Where applicable dephosphorylation was performed on the PVDF membrane with FastAP for 60 minutes at 37*C after blocking. The blocked membrane was incubated with primary antibodies O/N on a rotor at 4°C followed by washing three times 15 minutes in TBS-T on a shaker. Membranes were re-blocked for 15 minutes in either 5% skimmed milk powder or BSA on a shaker. Membranes incubated for 2 hours with secondary HRP-conjugated antibodies

on a shaker. Membranes were washed three times 15 minutes on a shaker with TBS-T before membranes were developed on X-ray sensitive films (Pierce, Cat#PI34091) using Western Lightning Plus-ECL substrate (PerkinElmer) in a dark room. Western blots were analysed using Image Studio (LI-COR Biosciences, Cat#RRID:SCR_015795) and data was visualized with Prism software (Graphpad, Cat#RRID:SCR_002798).

CIP2A phosphorylation was determined using a custom CIP2A phosphorylation specific antibody (GL Biochem) raised against a 10 amino acid phosphorylated peptide (I-N-P-E-T-V-N-L-(pS)-I – COOH) of CIP2A's extreme C-terminus. Total protein levels were determined with antibodies of CIP2A C-terminal (Santa Cruz, Cat#sc-80659), CIP2A N-terminal (Cell Signalling, Cat#14805), Actin (Sigma, Cat#A5316), GST (Santa Cruz, S Cat#sc-138), Pan14-3-3 (Santa Cruz, Cat#sc-133233).

GST14-3-3η protein production and purification

The expression vector pGex-2T-Hs 14-3-3η was a kind gift from Dr. J. D. Martinez, Arizona Cancer Center, Department of Cell Biology and Anatomy, The University of Arizona, Tucson, AZ, USA. Rosetta 2 (DE3)pLysS *E. coli* transformed with the pGex-2T-Hs 14-3-3η plasmid was grown in TB medium with 50 µg/mL of Ampicillin at 37°C until a OD_{600} of 0.6 was reached, protein expression was then induced with 0.4 mM of ITPG for 16 hours at 24*C shaking at 220 rpm. Bacteria were pelleted and subsequently resuspended in lysis buffer (300 mM NaCl, 50 mM Tris-HCL, 2 mM Dithiothreitol, 1 mg/mL Lysozyme, 10 µg/mL DNAseI, Glycerol 10% and complete protease inhibitor cocktail Roche; Cat# 11697498001). Cells were lysed by passage through an emulsifier and cell debris was removed by ultra-centrifugation at 100 000 g for 30 minutes. Protein purification was performed on an AKTÄ FPLC using GSTrap HP columns (GE Healthcare; Cat#GE17-5281-01).

GST-14-3-3η pulldowns

MDA-MB-468 (ATCC, Cat#HTB-132) cells were grown and harvested as described above, in the case of FC-A samples cells were grown the presence of 30 µM of FC-A or 0.15% DMSO for 24 hours before harvest. A total of 1.5 mg of cell lysate was incubated with 200 µM ATP, 10 mM $MgCl_2$ and or 100 µM N-terminally biotinylated Difopein peptide (Bio-S-A-D-G-A-P-H-C-V-P-R-D-L-S-W-L-D-L-E-A-N-M-C-L-P-G-A-A-G-L-D-S-A-D-G-A-P-H-C-V-P-R-D-L-S-W-L-D-L-E-A-N-M-C-L-P-G-A-A-G-L-E) and GST-14-3-3η for 1 hour at 37°C in a shaker. Magnetic GST beads (Genscript, Cat#L00327) were washed three times with PBS (pH=7.5) and 250 µL of beads was incubated with pulldown samples at 4°C O/N on a rotor. Supernatant was removed and beads were washed three times with 750 µL of PBS (pH=7.5) on a rotor for 5 minutes. Samples were eluted by incubation with 100 µL GST elution buffer (50 mM Tris + 50 mM L-Gluthathione reduced (Sigma, Cat#G4251), pH=8.0) on a rotor for 30 minutes. Samples analysed by western blot.

Quantification and statistical analysis

Statistical tests were performed using Prism 6.0 (Graphpad, Cat#RRID:SCR_002798). The following statistical tests, as indicated in the figure legends, were used: an unpaired t-test corrected for multiple

comparisons with the Holm-Sidak method, one way ANOVA with a Dunnett's multiple comparison test. Sample sizes are indicated in figure legends and significance was defined as *$p<0.05$, NS=not significant.

Data and code availability

The x-ray crystal structures generated in this study have been deposited to the Protein Data Bank (PDB codes: 7BM9 and 7BMC).
